# Fine‐tuning the artificial intelligence experience in endoscopy

**DOI:** 10.1002/ueg2.12253

**Published:** 2022-05-20

**Authors:** Keith Siau, Tyler M. Berzin

**Affiliations:** ^1^ Department of Gastroenterology Royal Cornwall Hospitals NHS Trust Truro UK; ^2^ Medical and Dental Sciences University of Birmingham Birmingham UK; ^3^ Center for Advanced Endoscopy Beth Israel Deaconess Medical Center and Harvard Medical School Boston Massachusetts USA

**Keywords:** artificial intelligence, colonoscopy, endoscopy, polypectomy

Artificial intelligence (AI) represents a transformative advance in the practice of gastrointestinal endoscopy, with giant strides already made in a short space of time. In the last 5 years alone, the publication of a number of high‐profile randomised trials now give a glimpse of endoscopy's symbiotic future with AI. Computer‐aided detection (CADe) technology has been shown to increase adenoma detection rates,^1^, ^2^ thereby reducing lesion miss rates,^3^ whereas computer‐aided diagnosis can accurately predict histology to guide real‐time management,^4^ potentially limiting unnecessary resection and histological analysis of hyperplastic lesions, with subsequent benefits on healthcare costs and endoscopy's carbon footprint.^5^ Additionally, AI applications offer potential for automated quality control systems to generate user prompts to modify behaviour in real‐time, for example, slowing down withdrawal and encouraging longer inspection and cleansing of poorly visualised areas.^6^

AI technologies which support a competent endoscopist to detect and diagnose polyps at the level of an expert are now already being installed in an increasing number of endoscopy units worldwide. However, despite the abundance of AI technologies in CADe and CADx, there are still opportunities to refine the capabilities of AI to provide further benefit to users.

In this issue,^7^ Brand et al perform a prospective multicentre study aimed to apply machine learning to minimise distractions from CADe systems during colonoscopy interventions, specifically AI alert boxes triggered by passage of a device into the field of view. The authors enrolled 580 procedures of which 8.8% contained visible instruments, and developed a convolutional neural network to block out unnecessary CADe triggers when biopsy forceps or snare was applied to an already detected lesion. This accommodated a variety of endoscopic processor systems (Olympus and Pentax) and was found to be accurate (sensitivity and specificity both >98.5%) for detecting devices, but also reduced potentially disrupting CADe triggers by 95.6%.

While polyp detection has been the ‘tip of the spear’ for AI in gastroenterology, this paper gives a nice glimpse of some of the broader capabilities of AI which will impact our practice in the future. The concept of whether false positive alerts during AI polyp detection could cause distractions (or other unintended consequences) at certain moments is an important one that should be a topic for future study. Some would question whether the efforts taken by the authors to automate CADe deactivation is truly necessary, especially when AI can be manually toggled on/off with a simple button press during instrument passage for polypectomy. However, the concept of automatically disabling AI is appealing and forms the basis for future development. There is still a technical leap between ‘recognizing’ the instrument using computer vision, and actually integrating this into the user experience practically during colonoscopy with AI polyp detection. For instance, does the presence of an instrument on the screen toggle the AI off for 5 s, 10 s, or on a frame‐by‐frame basis, which would be annoying for the user, if the AI blinks on and off depending on how clearly the instrument is seen on the screen? These questions, which are centred on ‘user interface’ design, are critical to address for such technology to be practical.

This study raises important questions and future research priorities. First, what is the best design for visual +/− auditory alarms to minimize alert fatigue (and distractions) while maximizing support for physicians? Second, as additional AI tools come along in future, how should complex AI‐generated data be presented without clogging up the endoscopy monitor and causing cognitive load? Thoughtful user interface design for AI systems in endoscopy will be critical so that physicians can integrate complex data during live endoscopy, without being distracted from the task at hand.

An AI tool to recognize endoscopic instruments could have several applications beyond just auto‐disabling polyp detection. Auto‐population of endoscopy reports is perhaps the most exciting avenue, especially if specific instruments and interventions can be recognized and documented with high accuracy. Radio‐frequency identification embedded instruments combined with AI recognition of interventions might offer a particularly robust approach to procedure reporting along with device invoicing. This could help deliver an AI process stream, whereby lesions can be detected, characterised, followed by documentation of tools used (e.g. snares, biopsy forceps etc) and their therapies, which could all be compiled within the endoscopy report. If accurately done, this could not only help reduce the overwhelming burden of electronic documentation for physicians, but can improve quality and consistency of procedure documentation, and performance metric reporting.

The AI endoscopy interface of the next decade will represent the culmination of innumerable small but important steps. The effort by Brand et al. gives a glimpse of the incremental innovations which will be a critical part in our AI journey for gastrointestinal endoscopy.
